# RecET driven chromosomal gene targeting to generate a RecA deficient *Escherichia coli *strain for Cre mediated production of minicircle DNA

**DOI:** 10.1186/1472-6750-6-17

**Published:** 2006-03-10

**Authors:** Oleg Tolmachov, Iwona Palaszewski, Brian Bigger, Charles Coutelle

**Affiliations:** 1 Section of Molecular and Cellular Medicine, Division of Biomedical Sciences, Faculty of Natural Sciences, Imperial College London, London SW7 2AZ, UK

## Abstract

**Background:**

Minicircle DNA is the non-replicating product of intramolecular site-specific recombination within a bacterial minicircle producer plasmid. Minicircle DNA can be engineered to contain predominantly human sequences which have a low content of CpG dinucleotides and thus reduced immunotoxicity for humans, whilst the immunogenic bacterial origin and antibiotic resistance marker gene sequences are entirely removed by site-specific recombination. This property makes minicircle DNA an excellent vector for non-viral gene therapy. Large-scale production of minicircle DNA requires a bacterial strain expressing tightly controlled site-specific recombinase, such as *Cre *recombinase. As recombinant plasmids tend to be more stable in RecA-deficient strains, we aimed to construct a *recA*^- ^bacterial strain for generation of minicircle vector DNA with less chance of unwanted deletions.

**Results:**

We describe here the construction of the RecA-deficient minicircle DNA producer *Escherichia coli *HB101Cre with a chromosomally located *Cre *recombinase gene under the tight control of the *araC *regulon. The *Cre *gene expression cassette was inserted into the chromosomal *lacZ *gene by creating transient homologous recombination proficiency in the *recA*^- ^strain HB101 using plasmid-born *recET *genes and homology-mediated chromosomal "pop-in, pop-out" of the plasmid pBAD75Cre containing the *Cre *gene and a temperature sensitive replication origin. Favourably for the *Cre *gene placement, at the "pop-out" step, the observed frequency of RecET-led recombination between the proximal regions of homology was 10 times higher than between the distal regions. Using the minicircle producing plasmid pFIXluc containing mutant *loxP66 *and *loxP71 *sites, we isolated pure minicircle DNA from the obtained *recA*^- ^producer strain HB101Cre. The minicircle DNA preparation consisted of monomeric and, unexpectedly, also multimeric minicircle DNA forms, all containing the hybrid *loxP66/71 *site 5'-TACCGTTCGT ATAATGTATG CTATACGAAC GGTA-3', which was previously shown to be an inefficient partner in Cre-mediated recombination.

**Conclusion:**

Using transient RecET-driven recombination we inserted a single copy of the *araC *controlled *Cre *gene into the *lacZ *gene on the chromosome of *E. coli recA*^- ^strain HB101. The resultant *recA*^- ^minicircle DNA producer strain HB101Cre was used to obtain pure minicircle DNA, consisting of monomeric and multimeric minicircle forms. The obtained *recA*^- ^minicircle DNA producer strain is expected to decrease the risk of undesired deletions within minicircle producer plasmids and, therefore, to improve production of the therapeutic minicircle vectors.

## Background

Plasmid DNA is recognised as foreign in vertebrate cells because of the abundance and the unmethylated status of CpG dinucleotides in the bacterial sequences. However, bacterially derived non-replicating minicircle DNA, which is created by removal of plasmid backbone DNA through intramolecular site-specific recombination within a minicircle producer plasmid, can be engineered to contain predominantly human sequences with a low incidence of immunostimulatory CpG-containing motifs [1]. Therefore, minicircle DNA is an excellent vector for non-viral gene therapy, where a robust immune response can interfere with gene delivery (reviewed in [2]). Large-scale production of minicircle DNA requires a bacterial strain containing a tightly controlled gene for site-specific recombinase, which can be expressed for production of the non-replicating minicircle after the bacterial culture harbouring the minicircle producer plasmid reaches the stationary growth phase. Previously we described the generation of a *recA*^+ ^*Escherichia coli *strain, MM219Cre, containing the chromosomally located Cre recombinase gene under the tight control of the *araC *regulon [3]. A single copy of the *Cre *gene ensures an extremely low leakage level of Cre recombinase expression under repression conditions. Untimely Cre recombinase expression during expansion of bacterial culture can cause maintenance instability of the minicircle producer plasmids and their premature dissociation into the minicircle and the miniplasmid recombination products with the subsequent loss of the non-replicating minicircle [4].

Low mutation load is of utmost importance in the production of DNA for human gene therapy protocols. RecA-deficient bacterial strains are often advantageous for production of plasmids with eukaryotic DNA sequences because undesirable spontaneous deletion mutations tend to occur with a lower frequency in the RecA-deficient background [5-7]. Therefore, we undertook to construct a RecA-deficient single-copy *Cre *gene expression system for production of the minicircle vector DNA. To insert the *Cre *gene into the bacterial chromosome we designed a version of the technique for gene placement, which relies on homology-mediated "pop-in, pop-out" of a plasmid with a temperature sensitive mutation in the pSC101 replication origin [8]. We employed a portable plasmid-born RecET recombination module [9] to create transient homologous recombination proficiency in the *recA*^- ^*E. coli *strain HB101, thus enabling use of "homology arms" to target an insertion of an expression cassette for the *araC *controlled *Cre *gene into the chromosomal *lacZ *gene.

## Results

### Integration ("pop-in") of plasmid pBAD75Cre into the genome of *E. coli *HB101

**Figure 1:**
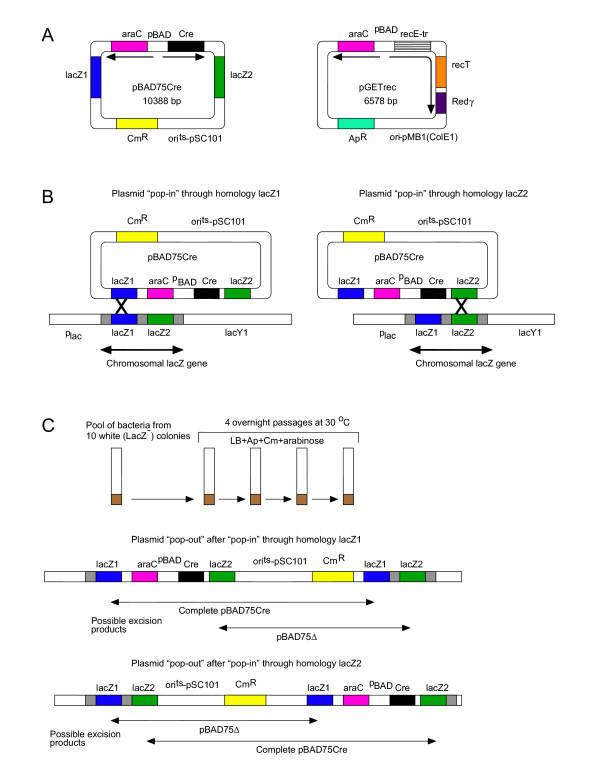
**Strategy to insert the *araC-Cre *cassette into the chromosomal *lacZ *gene in *recA13 *strain HB101**. **(A) **Compatible plasmids pBAD75Cre and pGETrec were introduced into *E. coli *HB101. The plasmid pBAD75Cre contains *araC-Cre *arabinose-inducible expression cassette flanked by targeting homologies to the *lacZ *gene. The plasmid pGETrec contains *araC-recET-Redγ *arabinose-inducible expression cassette comprising a truncated *recE *gene, the *recT *gene and the bacteriophage λ *Redγ *gene. **(B) **Expression of the *recET *genes was induced by L-arabinose to enable homologous recombination. Clones with integrations of the pBAD75Cre plasmid into the chromosomal *lacZ *gene were selected as white LacZ^- ^colonies on LB agar supplemented with Ap, Cm, X-gal and IPTG at 44°C. **(C) **Ten LacZ^- ^colonies were pooled and used for 4 overnight passages in L-arabinose containing medium (dilution 1:5000 at each passage step). Induction of *recET *expression by L-arabinose enabled excision of the entire pBAD75Cre or its deletion derivative pBAD75Δ through homologous recombination. Excision of pBAD75Δ resulted in the desired bacterial strain HB101Cre.

The homologous recombination deficient *E. coli recA13 *strain HB101 was transformed with the compatible plasmids pBAD75Cre and pGETrec (Figure 1A). The first plasmid, pBAD75Cre, provides a *Cre *expression cassette containing the *araC *regulator gene, which is flanked by two regions of homology to the bacterial *lacZ *gene to enable chromosomal insertion of the cassette via homologous recombination [3]. The plasmid also contains the mutant temperature sensitive replicon from the plasmid pSC101, which drives plasmid replication at 30°C, but is shut down in over 99% of cells at 44°C. The second plasmid, pGETrec [9], is a mobile recombination module with the truncated *recE *gene and the *recT *gene under control of the *araC *regulon. RecET recombinase can drive efficient homologous recombination in a RecA^- ^environment [10].

Expression of the *recET *genes was induced by L-arabinose. Subsequent plating of 2 × 10^4 ^HB101 cells harbouring both pBAD75Cre and pGETrec on LB agar containing X-gal, IPTG, Cm and Ap at 44°C enabled us to isolate 10 white LacZ^- ^clones containing an insertion ("pop-in") of the entire pBAD75Cre plasmid in the bacterial *lacZ *gene (Figure 1B).

To compare the details of our RecET-driven *Cre *gene placement procedure to a RecA-driven gene placement protocol described by Hamilton *et al*. [8], we introduced the targeting plasmid pBAD75Cre into *recA*^+ ^*E. coli *strain TB1 harbouring plasmid pUC19. The chromosomal integration target in this case was the *lacZΔM15 *gene expressing a truncated version of β-galactosidase protein, which attained full enzymatic activity in the presence of pUC19 expressing complementary α-peptide. After plating 1 × 10^4 ^cells on X-gal indicator plates at 44°C we isolated 7 white LacZ^- ^"pop-in" clones (see Additional file 1).

Insertions of the *ori*_*pSC*101_-plasmids in the bacterial genome often result in poor growth of the bacteria, thus, simplifying subsequent selection of the clones that underwent excision of the plasmid backbone [8]. We duly observed reduction in colony size of the obtained pBAD75Cre insertion derivatives of the *recA*^+ ^strain *E. coli *TB1. However, the colony size of the pBAD75Cre insertion derivatives of the *recA*^- ^strain *E. coli *HB101 remained unchanged compared to the original strain HB101. Our direct measurements of OD_600 _of the growing liquid bacterial cultures confirmed lack of significant differences between the growth rates of the HB101-based strains.

### Excision ("pop-out") of the pBAD75Cre plasmid backbone

To avoid the potential problem of spurious recombinants, the 10 LacZ^- ^insertion clones were pooled. The pool was passaged 4 times in LB medium supplemented with Ap, Cm, L-arabinose at 30°C to encourage plasmid excision ("pop-out") from the bacterial chromosome. The desired excision of the deletion derivative pBAD75Δ leaves the *araC-Cre *cassette on the chromosome whilst excision of the entire pBAD75Cre plasmid reconstitutes the intact chromosomal *lacZ *gene (Figure 1C). To confirm the presence of the LacZ^+ ^class of recombinants that underwent excision of the entire pBAD75Cre plasmid, we plated the passaged cells to produce individual colonies on LB agar supplemented with Cm, Ap, X-gal and IPTG at 30°C. Five LacZ^+ ^blue clones were found among 7511 analysed colonies (6.7 × 10^-4^).

**Figure 2:**
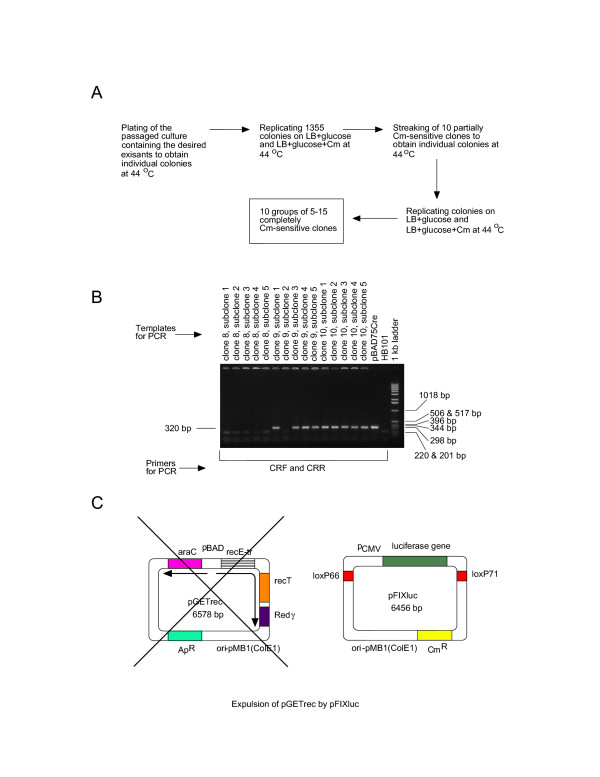
**Selection of the bacterial excisants with the *araC-Cre *insert in the chromosomal *lacZ *gene**. **(A) **The passaged bacterial population, which contained the excisants, was plated at 44°C to obtain individual colonies and to encourage the loss of the excised plasmids. Screening revealed 10 partially Cm-sensitive clones, which were proven to be mixtures of Cm-sensitive (plasmidless) and Cm-resistant (plasmid-bearing) cells. Subcloning resulted in isolation of completely Cm-sensitive plasmidless clones. **(B) **The obtained Cm-sensitive subclones were screened for the presence of the *Cre *gene by PCR using primers CRF and CRR homologous to the *Cre *gene. Bacteria were added directly to the PCR reaction mixtures. **(C) **Finally, the unwanted plasmid pGETrec was expelled from the *Cre*-positive clones by the incompatible minicircle producer plasmid pFIXluc maintained by selection for Cm-resistance.

The clones with excised plasmids should acquire inability to grow on selective agar with Cm at 44°C because of the loss of *ori*^*ts*^_*pSC101*_-plasmids at this temperature. To find the clones with "popped-out" plasmids, Hamilton *et al*. produced individual colonies from the passaged culture at 30°C and screened for Cm-sensitivity at 44°C [8]. In our preliminary experiments such screening was difficult to perform because of the substantial background of cells with replicating *ori*^*ts*^_*pSC101*_-plasmids at 44°C. Therefore, we plated the passaged cell population at 44°C to produce individual colonies in conditions favouring loss of the *ori*^*ts*^_*pSC101*_-plasmids. Screening of 1355 individual colonies (also at 44°C) did not reveal any outright Cm-sensitive clones but 10 partially Cm-sensitive clones were spotted (7.4 × 10^-3^) and re-streaked to obtain individual colonies, which were used to screen again for Cm-sensitivity. Between 5 and 15 completely Cm-sensitive subclones were isolated for each of the 10 clones (Figure 2A).

The obtained Cm-sensitive subclones were checked for their LacZ phenotype on the indicator plates containing LB agar supplemented with IPTG and X-gal, and for the presence of the *Cre *gene by PCR (Figure 2B). On this occasion we did not find any LacZ^+ ^subclones in which the entire plasmid has been excised and the *lacZ *gene resealed. All tested Cm-sensitive sublcones were LacZ^- ^suggesting an insertion in the chromosomal *lacZ *gene. However, according to PCR analysis, 5 partially Cm-sensitive clones produced only *Cre*-positive subclones, 4 clones produced a mixture of *Cre*-positive and *Cre*-negative subclones and 1 clone gave only *Cre*-negative subclones. In total, among 96 tested subclones the *Cre *gene was lost in 32 (33%). LacZ^- ^*Cre*^- ^segregants were probably due to plasmid excisions via illegitimate recombination resulting in deletions.

A similar plasmid "pop-out" strategy was followed with the pBAD75 integration derivatives of the control *recA*^+ ^*E. coli *strain TB1 (see Additional file 2). Markedly higher frequencies of excisant recovery were obtained. LacZ^+ ^excisants constituted 2.5 × 10^-2 ^of the passaged cell population (31 out of 1245). Screening at 44°C allowed to find 21 partially Cm sensitive clones out of 500 (4.2 × 10^-2^), 9 of which were proved to be LacZ^+ ^*Cre*-negative, 11 clones were LacZ^- ^*Cre*-positive and 1 clone produced a mixture of LacZ^+ ^*Cre*^-^, LacZ^- ^*Cre*^- ^and LacZ^- ^*Cre*^+ ^subclones.

**Figure 3:**
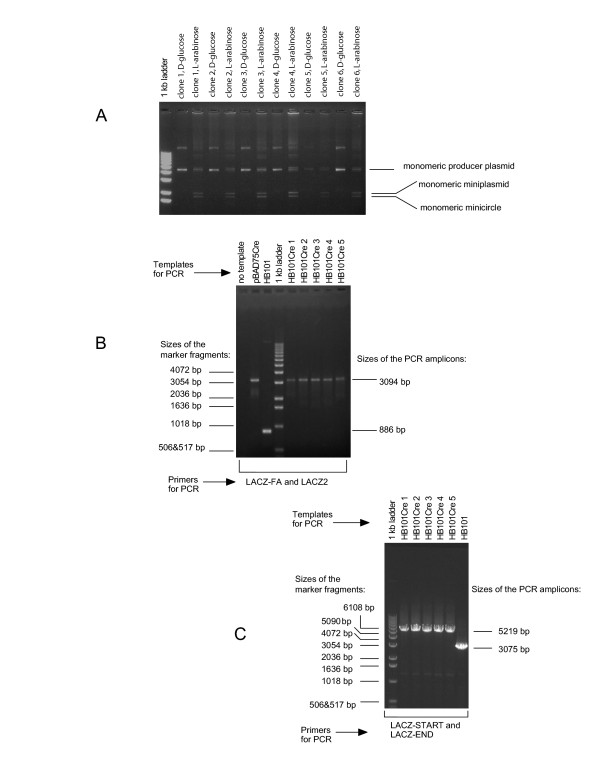
**Minicircle DNA production test and long range PCR analysis of HB101Cre clones**. **(A) **Minicircle DNA production test. Cultures of the prospective HB101Cre strain harbouring minicircle producer plasmid pFIXluc were grown overnight in the LB medium supplemented with 0.2% D-glucose under Cm selection. The cells from each bacterial clone were washed in the minimal M9 medium, split into 2 tubes containing the M9 medium supplemented with 0.2% D-glucose (control) and 0.2% L-arabinose, cultured for 18 hrs and used for closed circular DNA extraction. The resultant DNA was studied by electrophoresis in a 0.7% agarose gel. Analysis for 6 clones is shown. **(B) **Long range PCR analysis to show successful introduction of the *araC-Cre *expression cassette into *E. coli *HB101. Primers LACZ-FA and LACZ2 were used to amplify the 3094 bp DNA fragment with the *araC-Cre *expression cassette. In all PCR analysis experiments bacteria were added directly to the PCR reaction mixtures and electrophoresis was performed in a 0.7% agarose gel. **(C) **Long range PCR analysis to show successful insertion of the *araC-Cre *cassette into the chromosomal *lacZ *gene. The *araC-Cre *segment replaced 886 bp of the *lacZ *gene. Primers LACZ-START and LACZ-END are homologous to the start and the end of the β-galactosidase coding sequence respectively. These primers were used to amplify the entire 5219 bp *lacZ::(araC-Cre) *fragment of the generated strain HB101Cre (the *lacZ *gene is 3075 bp).

To avoid bad luck of picking up a single *Cre*-positive subclone containing a pBAD-promoter re-arrangement, the *Cre*-positive subclones were pooled. The pooled cell population was transformed with the minicircle producer plasmid pFIXluc containing mutant *loxP66 *and *loxP71 *sites [3] in conditions of the *Cre *gene repression by D-glucose (Figure 2C). In the absence of selection by Ap, the *recET*-bearing plasmid pGETrec was rapidly lost because it was incompatible with pFIXluc, which was maintained by selection with Cm. We obtained 24 Ap-sensitive clones, grew bacterial cultures in the presence of D-glucose to repress the *Cre *gene, washed the cells and transferred them into the minimal medium supplemented with L-arabinose to induce Cre recombinase expression. Formation of the minicircle and the miniplasmid DNA was observed in all the tested clones, thereby confirming successful chromosomal placement of the *Cre *gene (Figure 3A). Complete expulsion of the *recET*-bearing plasmid pGETrec from several clones of the new strain HB101Cre pFIXluc was confirmed by PCR with primers AR1F and RE2R, which was specific for the junction between the plasmid-born *araC *and *recE *genes (see Additional file 3).

The inserted *araC-Cre *expression cassette and the entire bacterial *lacZ::araC-Cre *locus were amplified by long-range PCR, thus, proving successful insertion of the *araC-Cre *cassette into the *lacZ *gene of the strain HB101 (Figure 3BC). The wild type sequence of the inserted *Cre *gene was also confirmed (see Additional file 4).

The resultant strain HB101Cre pFIXluc was cured of the pFIXluc plasmid to allow unimpeded introduction of other minicircle producing plasmids. Finally, the RecA^- ^phenotype of the new strain HB101Cre was confirmed by the UV-sensitivity test [11].

### Production and isolation of minicircle DNA in *recA*^- ^bacterial strain HB101Cre

**Figure 4:**
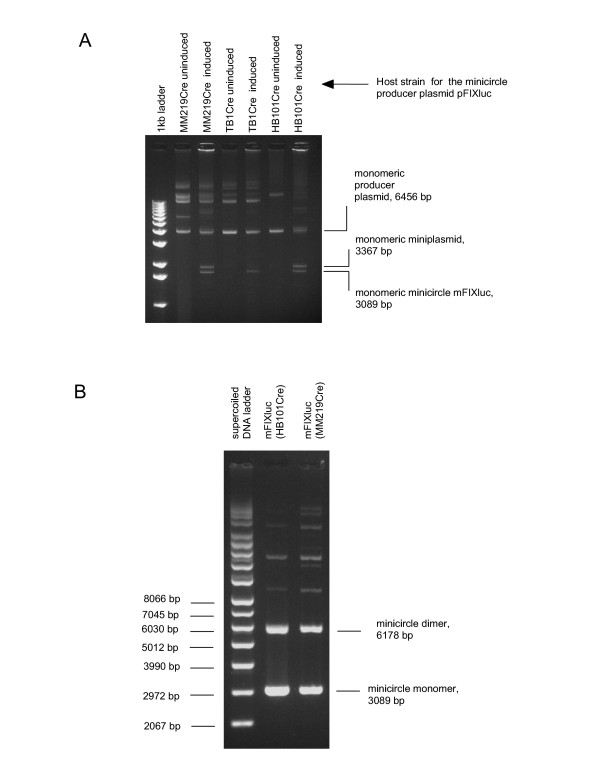
**Minicircle DNA production *in vivo***. Minicircle DNA was produced using producer plasmid pFIXluc. Electrophoresis was performed in a 0.7% agarose gel in the presence of ethidium bromide. **(A) **Analysis of the supercoiled DNA extracted from the *recA*^- ^strain HB101Cre, the *recA*^+ ^strain MM219Cre and the *recA*^+ ^strain TB1Cre, all harbouring pFIXluc, before and after induction of the *Cre *gene expression with L-arabinose. Intramolecular *Cre *recombination in the minicircle producer plasmid pFIXluc (6456 bp) gave rise, in particular, to the monomeric minicircle mFIXluc (3089 bp) and the monomeric miniplasmid (3367 bp). **(B) **Agarose gel analysis of the purified minicircle mFIXluc isolated from the *recA*^- ^strain HB101Cre pFIXluc and the *recA*^+ ^strain MM219Cre pFIXluc.

Six half litre cultures of the *recA*^- ^strain HB101Cre, six half litre cultures of the control *recA*^+ ^strain MM219Cre and one half litre culture of the control *recA*^+ ^strain TB1Cre, all of them harbouring the minicircle producer plasmid pFIXluc, were grown under catabolite repression conditions. The cells from each culture were washed, transferred to minimal medium supplemented with L-arabinose, cultured for additional 18 hrs and used to extract supercoiled DNA. Formation of the minicircle DNA was observed in all three bacterial strains (Figure 4A). The minicircle DNA was purified individually from each of the 6 HB101Cre-derived supercoiled DNA preparations and each of the 6 MM219Cre-derived preparations as described previously [3]. The minicircle DNA yields were 248 ± 25 μg per half a litre culture for HB101Cre strain and 225 ± 18 μg per half a litre culture for MM219Cre strain. Surprisingly, there was no considerable difference in the extent of multimerisation in HB101Cre-extracted and MM219Cre-extracted minicircle DNA (Figure 4B). The HB101Cre-produced and the MM219Cre-produced minicircle DNA preparations were used for sequencing through the junction formed by intramolecular Cre recombination. The expected sequence of the newly emerged hybrid site *loxP66/71 *(5'-TACCGTTCGT ATAATGTATG CTATACGAAC GGTA-3') and the adjacent sequences were confirmed in both minicircle DNA preparations (see Additional file 5).

## Discussion

The minicircle DNA is an attractive "stealth vector" for human gene therapy because it can be engineered to consist virtually entirely of mammalian sequences with low occurrence of CpG motifs that are recognised as a potent signal by the innate immune system. Plasmid DNA for human use is normally produced in *recA*^- ^bacterial strains [7] because the rate of deletion mutations in recombinant plasmids in RecA-deficient environment is often lower than in RecA-proficient environment [5,6]. We describe here an upgrade of our Cre-mediated system for minicircle DNA production [3] to a *recA*^- ^status.

The described method of chromosomal gene placement is an adaptation of the method of Hamilton *et al*. [8] to the situation where the bacterial strain to be manipulated is RecA-deficient. The major technical difficulty both in the original version of the procedure and its adaptation in our work is negative selection of bacterial clones with resolved plasmid-chromosome co-integrates. Clearly, any selective pressure towards clones with popped-out plasmids simplifies the required screening. Fortuitously for their experiments, Hamilton *et al *observed, "this method works because the presence of the pSC101 replication origin in the *E. coli *chromosome dramatically reduced the growth rate of the cell; pSC101 co-integrates grew very poorly at 30°C on L-agar containing chloramphenicol". In our experiments we confirmed this crucial growth retardation phenomenon for the control *recA*^+ ^strain *E. coli *TB1 and pSC101-based plasmid pBAD75Cre. In this case we were able to obtain plasmid pop-out clones with a comfortable frequency of 4.2 × 10^-2^. However, we did not detect any growth retardation for the bacterial clones harbouring co-integrates of the *recA*^- ^*E. coli *HB101 chromosome and pBAD75Cre. Still we were able to recover plasmid pop-out clones, although with a lower frequency of 7.4 × 10^-3^.

Our intention to generate a bacterial strain for production of therapeutic minicircle DNA limited the scope of available strategies to achieve the desired combination of a chromosomally located *Cre *gene and the *recA *mutation. For example, the required *Cre*-expressing *recA*^- ^strain could have been created by the exchange of the *recA*^+ ^allele for a *recA*^- ^allele in the previously constructed *Cre*-expressing strain MM219Cre [3] through generalised transduction by the bacteriophages P1 or T4GT7 from a *srl::Tn10 recA *strain [12]. However, the presence of Tn10 in the minicircle DNA producer strain is undesirable since some human therapeutic genes contain potential mutational hotspots for this element (5'-GCTNAGC-3' [13]). Thus, cDNA for the human CFTR gene, the therapeutic gene for cystic fibrosis, has one hotspot consensus sequence and cDNA for the human dystrophin gene, the therapeutic gene for DMD, has three such potential hotspots. Similarly, we wished to avoid methods of chromosomal gene placement that directly employ insertions by transposons, bacteriophage Mu or IS-elements [14]. The remaining options for construction of the new *recA*^- ^strain included hijacking of the site-specific chromosomal integration systems of the bacteriophages λ, φ80 and P2, and also homology-mediated gene targeting enabled by introduction of RecA [15], RecET or Redαβ-expressing plasmids.

Here we describe the construction of the *recA*^- ^minicircle DNA producer *E. coli *HB101Cre using transient homologous recombination proficiency created by the plasmid-born *recET *genes and homology-mediated chromosomal "pop-in, pop-out" of a plasmid with a temperature sensitive replication origin, pBAD75Cre.

It is not clear why the observed frequency of recombination leading to the excision of the entire pBAD75Cre plasmid from the *lacZ *gene (5 LacZ^+ ^excisants out of 7511 colonies, 6.7 × 10^-4^) was 10 times lower than the frequency of recombination leading to the excision of the plasmid backbone pBAD75Δ, which left the *araC-Cre *expression cassette in the bacterial chromosome (9 LacZ^- ^*Cre*-positive individual excisants out of 1355 colonies, 6.6 × 10^-3^). Using homologous recombination driven by the plasmid-expressed RecA protein, some authors observed a strong bias towards one or the other type of "pop-out" recombination among individual plasmid insertions but did not notice any common predisposition among all the plasmid insertions [16]. We did not observe any significant disproportion between recombination classes in RecA-driven plasmid pop-out in the control strain TB1. Screening of 500 colonies yielded 11 LacZ^+ ^*Cre*^- ^(2.2 × 10^-2^) and 9 LacZ^- ^*Cre*^+ ^clones (1.8 × 10^-2^). Therefore, we hypothesise that predominant recombination between two proximal regions of homology (compared to recombination between two distal regions of homology) can be a feature of RecET-driven recombination. If this is indeed so, this property of RecET recombination is extremely advantageous for the selection of insertions into the bacterial chromosome or large plasmids after the "pop-out" step of the "pop-in, pop-out" scheme of recombinogenic engineering ([17], reviewed in [18]).

Using the new *recA*^- ^strain HB101Cre we obtained a yield of 248 ± 25 μg of pure minicircle DNA per half a litre of bacterial culture and observed formation of minicircle multimers.

The *recA13 *mutation in the HB101Cre strain inhibits homologous recombination. At the same time, the hybrid *loxP66/71 *site, which is present on the minicircle mFIXluc, contains 10 nucleotide substitutions in the 34 bp sequence of the wild type *loxP *site and is known to be a poor substrate for Cre recombination [19]. Therefore, formation of the minicircle multimers in the HB101Cre bacteria, containing the wild type *Cre *gene, does not fit well both the homologous recombination mechanism of multimerisation and the Cre-mediated recombination mechanism of multimerisation [4].

Formation of the *loxP66/71 *minicircle multimers in a *recA*^- ^strain can be attributed to induction of the resident chromosomal *recET *genes [20] or induction of an additional *recA*-independent pathway, such as discovered recently [21]. Recombinant plasmids are known to change host gene expression patterns in *E. coli *[22] and so, the minicirlcle producer plasmid pFIXluc might have been the trigger for induction of the endogenous recombination genes in our bacterial strain HB101Cre.

There are no data to suggest that the multimeric minicircle forms are inferior to the minicircle monomer in terms of gene delivery to mammalian cells. In fact, the multimeric minicircle forms might be of some benefit for higher levels of transient transgene expression [23,24].

## Conclusion

In summary, we inserted the *araC-Cre *gene expression cassette into the chromosomal *lacZ *gene of *E. coli recA*^- ^strain HB101 by creating transient homologous recombination proficiency using the plasmid-born *recET *genes and employing homology-mediated chromosomal "pop-in, pop-out" of a *Cre *plasmid pBAD75Cre with a temperature sensitive replication origin. Favourably for the *Cre *gene placement, at the "pop-out" step, the observed frequency of RecET-led recombination between the proximal homology regions was 10 times higher than that between the distal homology regions. We isolated pure minicircle DNA, which was generated in RecA-deficient background, and showed the presence of the multimeric minicircle forms containing mutant *loxP66/71 *site 5'-TACCGTTCGT ATAATGTATG CTATACGAAC GGTA-3', known to be a poor substrate for Cre-recombinase. Therefore, we question the view that Cre-mediated recombination is the dominant mechanism for formation of minicircle multimers. The obtained *recA*^- ^minicircle DNA producer strain is expected to decrease the risk of undesired deletions within minicircle producer plasmids and, therefore, to improve production of the therapeutic minicircle vectors.

## Methods

### *E. coli *strains and bacterial culture conditions

The following *E. coli *strains were used:

HB101 F^- ^*Δ(gpt-proA)62 leuB6 glnV44 ara-14 galK2 lacY1 Δ(mcrC-mrr) rpsL20(Str*^*R*^) *xyl-5 mtl-1 recA13 *(Promega);

HB101Cre F^- ^*Δ(gpt-proA)62 leuB6 glnV44 ara-14 galK2 lacZ::(araC Cre) lacY1 Δ(mcrC-mrr) rpsL20(Str*^*R*^) *xyl-5 mtl-1 recA13 *(this work);

MM219Cre F^- ^*endA1 hsdR17*(*r*_*K*_^-^*m*_*K*_^+^) *glnV44 thi-1 relA1? rfbD1? spoT1? lacZ::(araC Cre) *[3];

TB1 F^- ^*ara Δ(lac-proAB) [φ 80dlac:: Δ(lacZ)M15] rpsL(Str*^*R*^) *thi hsdR *(New England Biolabs),

TB1Cre F^- ^*ara Δ(lac-proAB) [φ 80dlac::(araC-Cre) Δ(lacZ)M15] rpsL(Str*^*R*^) *thi hsdR *(this work).

The bacteria were cultured as described before [3]. A *lacY *mutation in the strain *E. coli *HB101 results in a poor transport of the chromogenic substrate X-gal into the cells. Thus, for reliable identification of the LacZ^+ ^phenotype of the bacterial colonies, the sparsely seeded LB-agar indicator plates supplemented with X-gal and IPTG were incubated overnight at 37°C and then for additional 2–3 days at room temperature for the blue colour of the colonies to mature.

### Plasmids, oligonucleotides, long distance PCR and sequencing

Minicircle producer plasmid pFIXluc and the *Cre *gene containing plasmid pBAD75Cre were described previously [3]. Plasmid pGETrec [9] contains a truncated *recE *gene, the *recT *gene and the bacteriophage λ *Redγ *gene under control of the *araC *regulon.

Oligonucleotides CRF 5'-TGCCACGACCAAGTGACAGC AATG-3' and CRR 5'-CTCTACACCTGCGGTGCTAACCAGCG-3' were used to screen bacterial colonies for the presence of the *Cre *gene by PCR. Primers AR1F 5'-TTCCCAGCGG TCGGTCGATA AAAAAATCGA-3' and RE2R 5'-CAAGTACCCG GCAGTGGAAA GCAGTTCCTA-3' were used to amplify the 680 bp segment from the plasmid pGETrec containing pBAD promoter and a portion of the *recE *gene. The PCR in this case was led by Taq polymerase starting with 2 min at 93°C, followed with 30 cycles of 30 s at 93°C, 1 min at 58°C, 1 min at 72°C; and concluding with 7 min at 72°C.

The "Expand Long Template PCR" kit (Roche) and primers LACZ-FA 5'-CATGGTCAGGTCATGGATGAGCAGACG-3' and LACZ2 5'-GCGCCACCATCCAGTGCAGGAGCTCG-3' homologous to the *lacZ *gene were used to amplify the 3094 bp fragment with the *araC-Cre *insert in the *lacZ *gene of the HB101Cre and TB1Cre strains. The *Cre *gene within the amplified 3094 bp fragment was sequenced from both strands using primers ARAC-SEQ 5'-GATGGGCATT AAACGAGTAT CCCGGCAG-3', OPP-CRE 5'-GGGCACACAC TACTTGAAGC ACTC-3', CRF and CRR (Advanced Biotechnology Centre, Imperial College London). The same long distance PCR kit and primers LACZ-START 5'-ATGACCATGA TTACGGATTC ACTGGCCGTC GTTTT-3' and LACZ-END 5'-TTATTTTTGA CACCAGACCA ACTGGTAATG GTAGC-3' were used to amplify the entire 5219 bp *lacZ::(araC Cre) *genome segment of the HB101Cre strain. Long distance PCR algorithm was: 2 min at 93°C; 10 cycles of 10 s at 93°C, 30 s at 65°C and 4 min at 68°C; then 20 cycles at 10 s at 93°C, 30 s at 65°C, 4 min at 68°C with 20 s extension of the last (elongation) step in each new cycle; then 7 min at 68°C.

Primers SEQ-CMV 5'-ACATGGCGGT CATATTGGAC ATGAG-3' and SEQ-D 5'-CAATAAACAA GTTAACAACA ACAAT-3' were employed for the automated sequencing of both strands of the hybrid *loxP66/71 *site and its flanking regions in the minicircle mFIXluc.

### Minicircle DNA production

Induction of the *Cre *gene using L-arabinose, extraction of supercoiled DNA and minicircle DNA purification were performed essentially according to the previously described "Technique 2" [3] with two protocol changes. Firstly, expansion of the bacterial culture, which was destined for Cre induction, was performed at 32°C instead of 37°C, because this lower temperature was shown to support more consistent minicircle DNA yields (Thorsten Trimbuch, personal communication). Secondly, to improve resolution of the minicircle producer plasmid by the induced Cre recombinase in HB101Cre cells, incubation of bacteria with L-arabinose was performed for 18 hrs instead of 4–6 hrs (at 37°C, as described previously). Closed circular DNA was extracted by the alkaline method and digested with PvuII to fragment all non-minicircle products of Cre recombination. Finally, the supercoiled minicircle DNA was isolated using fractionation in CsCl-propidium iodide density gradient [3].

## List of abbreviations

Ap – ampicillin, CFTR – cystic fibrosis transmembrane conductance regulator, DMD – Duchenne muscular dystrophy, Cm – chloramphenicol, IPTG – isopropyl-D-thiogalactoside, OD600 – optical density measured at 600 nm, PCR – polymerase chain reaction, X-gal – 5-bromo-4-chloro-3-indolyl-β-D-galactopyranoside.

## Authors' contributions

OT conceived the study, performed most of the experiments, drafted and revised the manuscript. IP performed some of the experiments. BB and CC revised the manuscript. All authors read and approved the final manuscript.

## Supplementary Material

Additional File 1 **Strategy to insert the *araC-Cre *cassette into the chromosomal *lacZΔM15 *gene in *recA*^+ ^strain TB1**. Compatible plasmids pBAD75Cre and pUC19 were introduced into *E. coli *TB1 *lacZΔM15*. **(A) **The plasmid pBAD75Cre contains the *araC-Cre *arabinose-inducible expression cassette flanked by targeting homologies to the *lacZ *gene, which also fit a truncated *lacZΔM15 *version of the gene. The plasmid pUC19 contains a portion of the *lacZ *gene expressing α-peptide, which can bind the *lacZΔM15 *gene product and enable its β-galactosidase enzymatic activity. **(B) **Seven clones with integrations of the pBAD75Cre plasmid into the chromosomal *lacZΔM15 *gene were selected as white LacZ^- ^colonies on LB agar supplemented with Ap, Cm, X-gal and IPTG at 44°C. **(C) **Seven LacZ^- ^colonies were pooled and used for 4 overnight passages in LB medium supplemented with Ap and Cm (dilution 1:5000 at each passage step). Excision of the entire pBAD75Cre or its deletion derivative pBAD75Δ occurred via RecA-mediated homologous recombination. Click here for file

Additional File 2 **Selection of the bacterial excisants with the *araC-Cre *insert in the chromosomal *lacZΔM15 *gene**. **(A) **The passaged bacterial population, which contained the excisants, was plated at 44°C to obtain individual colonies and to encourage the loss of the excised plasmids. Screening of 500 colonies revealed 21 partially Cm-sensitive clones, which were proven to be mixtures of Cm-sensitive (plasmidless) and Cm-resistant (plasmid-bearing) cells. The obtained Cm-sensitive subclones were screened for the presence of the *Cre *gene by PCR using primers CRF and CRR homologous to the *Cre *gene sequence and primers LACZ-FA and LACZ2 homologous to the sequences flanking the *araC-Cre *DNA segment. Bacteria were added directly to the PCR reaction mixtures. **(B) **Finally, the unwanted plasmid pUC19 was expelled from the *Cre*-positive clones by the incompatible minicircle producer plasmid pFIXluc maintained by selection for Cm-resistance. Click here for file

Additional File 3 **Demonstration of eviction of the pGETrec plasmid from the strain *E. coli *HB101Cre pFIXluc**. A PCR test was performed to confirm complete eviction of the plasmid pGETrec from the clones of bacterial strain *E. coli *HB101Cre pFIXluc. Bacterial cells were directly added to the PCR mixtures containing primers AR1F and RE2R. **(A) **A diagram showing the PCR test strategy. **(B) **An agarose gel electrophoresis indicating absence of amplification of the pGETrec-specific 680 bp DNA segment after pGETrec eviction by the incompatible plasmid pFIXluc. Click here for file

Additional File 4 **Sequencing of the *Cre *gene from the bacterial strain *E. coli *HB101Cre**. Primers LACZ-FA and LACZ2 homologous to the *lacZ *gene were used to amplify the 3094 bp fragment with the *araC-Cre *insert in the *lacZ *gene of the HB101Cre. **(A) **A diagram showing the strategy to sequence the *Cre *gene within the amplified 3094 bp DNA fragment. Primers ARAC-SEQ, CRF, CRR and OPP-CRE (5'-GGGCACACAC TACTTGAAGC ACTC-3') were used to sequence the opposite DNA strands. The obtained sequence was shown to be identical to the wild type *Cre *sequence [GenBank:AF234172]. **(B) **A part of the electropherogram showing the sequence 5'-ACC CTG TTA CGT ATA GCC GAA ATT GCC AGG ATC AGG-3' of the *Cre *gene (obtained using primer CRF), which encodes a stretch of amino acids TLL**R**IAEIARIR including R173 (shown in bold) from the Cre recombinase catalytic site [25]. **(C) **A part of the electropherogram showing the same sequence as in **(B)**, read from the opposite strand (5'-CCT GAT CCT GGC AAT TTC GGC TAT ACG TAA CAG GGT-3', obtained using primer CRR). **(D) **A part of the electropherogram showing the sequence 5'-TCT GGA CAC AGT GCC CGT GTC GGA GCC GCG CGA GAT-3' of the *Cre *gene (obtained using primer CRF), which encodes a stretch of amino acids SG**H**SA**R**VGAARD including H289 and R292 (shown in bold) from the Cre recombinase catalytic site [25]. **(E) **A part of the electropherogram showing the sequence 5'-TGG ACC AAT GTA AAT ATT GTC ATG AAC TAT ATC CGT AAC-3' of the *Cre *gene (obtained using primer CRF), which encodes a stretch of amino acids **W**TNVNIVMN**Y**IRN including W315 and Y324 (shown in bold) from the Cre recombinase catalytic site [25]. Click here for file

Additional File 5 **Sequencing of the minicircle DNA**. Minicircle mFIXluc was isolated from the *recA*^- ^*E. coli *strain HB101Cre and *recA*^+ ^*E. coli *strain MM219Cre, both harbouring the minicircle producer plasmid pFIXluc before L-arabinose induction of Cre recombinase expression. **(A) **A diagram showing minicircle DNA sequencing strategy. Primers SEQ-CMV and SEQ-D were used to sequence opposite strands of the hybrid *loxP66/71 *site and the flanking regions. **(B) **A part of the electropherogram showing the sequence of *loxP66/71 *(5'-TACCGTTCGT ATAATGTATG CTATACGAAC GGTA-3') of the minicircle mFIXluc extracted from *E. coli *HB101Cre (SEQ-CMV primed extension). **(C) **A part of the electropherogram showing the sequence of *loxP71/66 *(5'-TACC GTTCGTATAG CATACATTAT ACGAACGGTA-3') of the minicircle mFIXluc extracted from *E. coli *HB101Cre (SEQ-D primed extension). **(D) **A part of the electropherogram showing the sequence of *loxP66/71 *site of the minicircle mFIXluc extracted from *E. coli *MM219Cre (SEQ-CMV primed extension). **(E) **A part of the electropherogram showing the sequence of *loxP71/66 *site of the minicircle mFIXluc extracted from *E. coli *MM219Cre (SEQ-D primed extension). Click here for file
