# Supplementary figures and images for: RecET driven chromosomal gene targeting to generate a RecA deficient Escherichia coli strain for Cre mediated production of minicircle DNA
Source: BMC Biotechnol. 2006 Mar 10;6:17. doi: 10.1186/1472-6750-6-17 (PMC1421399; doi:10.1186/1472-6750-6-17)

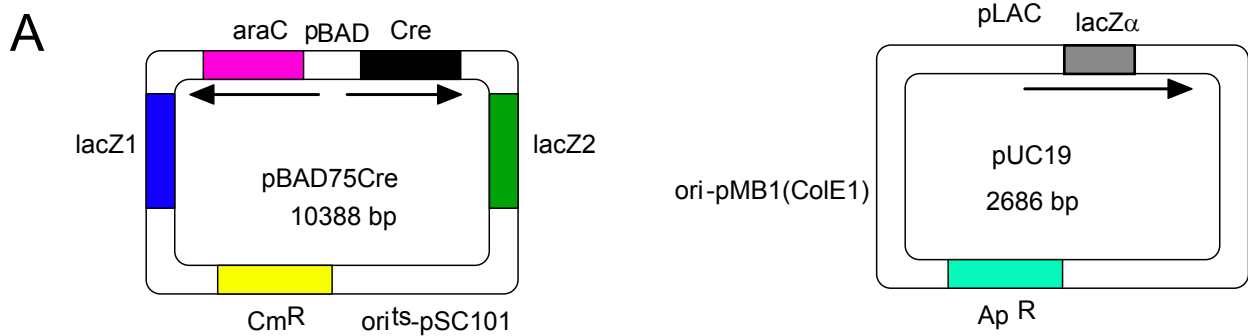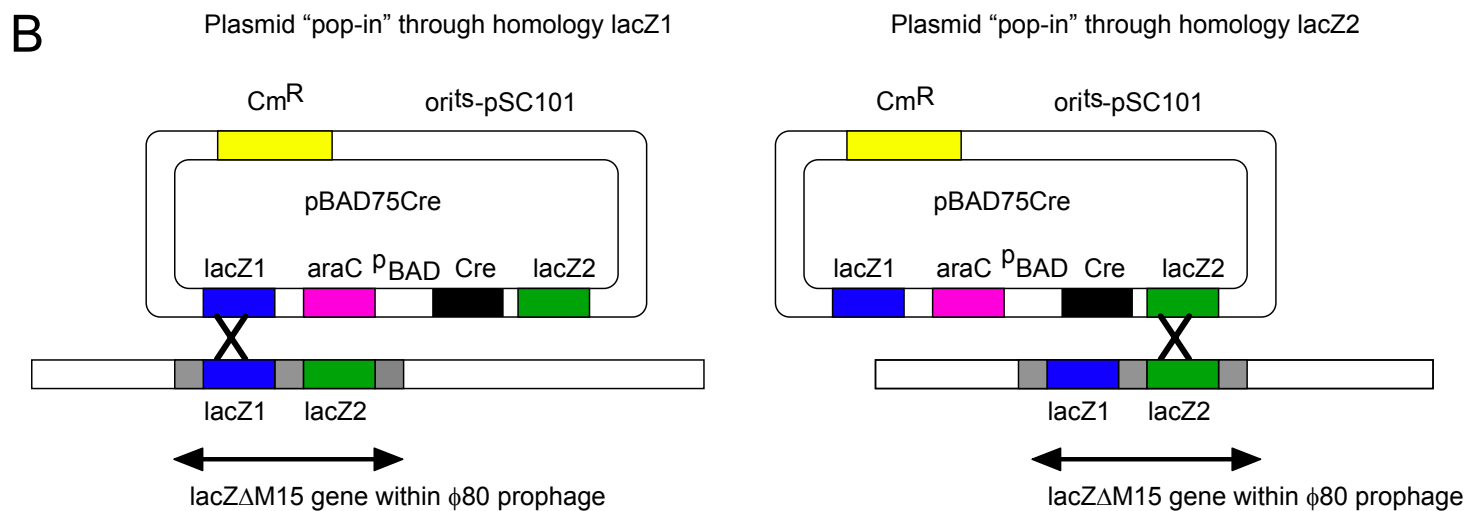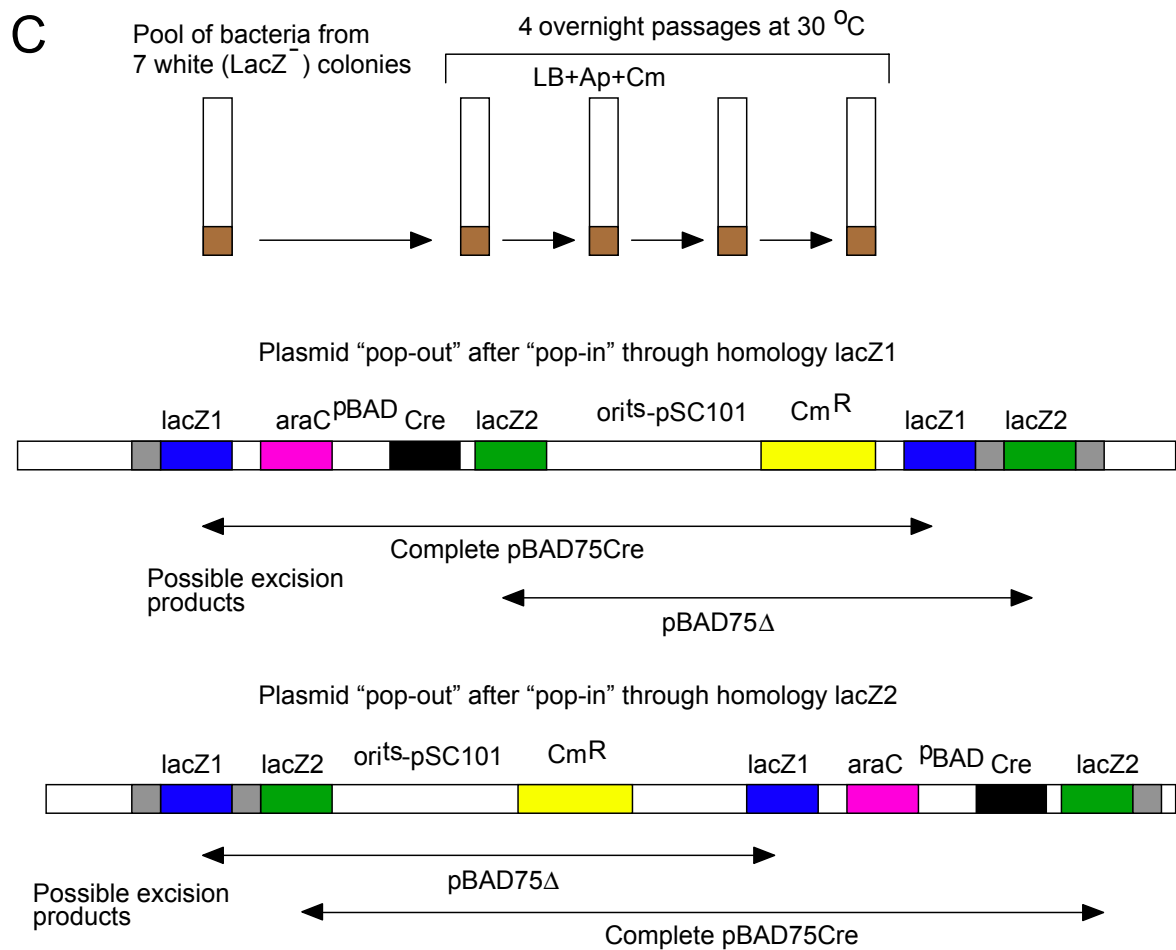

Supplement: Additional File 1 — Strategy to insert the araC-Cre cassette into the chromosomal lacZΔM15 gene in recA+ strain TB1. Compatible plasmids pBAD75Cre and pUC19 were introduced into E. coli TB1 lacZΔM15. (A) The plasmid pBAD75Cre contains the araC-Cre arabinose-inducible expression cassette flanked by targeting homologies to the lacZ gene, which also fit a truncated lacZΔM15 version of the gene. The plasmid pUC19 contains a portion of the lacZ gene expressing α-peptide, which can bind the lacZΔM15 gene product and enable its β-galactosidase enzymatic activity. (B) Seven clones with integrations of the pBAD75Cre plasmid into the chromosomal lacZΔM15 gene were selected as white LacZ- colonies on LB agar supplemented with Ap, Cm, X-gal and IPTG at 44°C. (C) Seven LacZ- colonies were pooled and used for 4 overnight passages in LB medium supplemented with Ap and Cm (dilution 1:5000 at each passage step). Excision of the entire pBAD75Cre or its deletion derivative pBAD75Δ occurred via RecA-mediated homologous recombination. [file 1472-6750-6-17-S1.pdf]

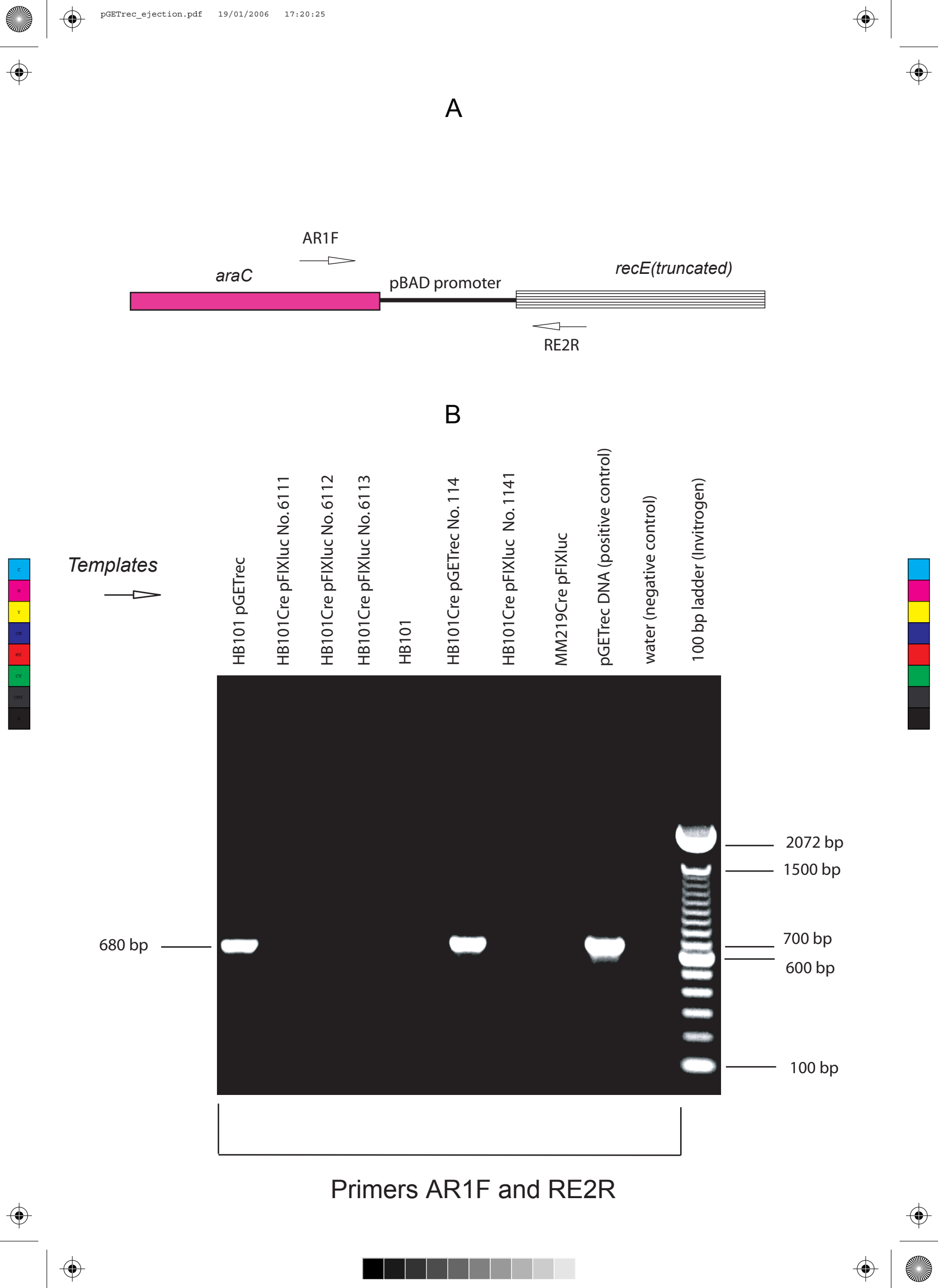

Supplement: Additional File 3 — Demonstration of eviction of the pGETrec plasmid from the strain E. coli HB101Cre pFIXluc. A PCR test was performed to confirm complete eviction of the plasmid pGETrec from the clones of bacterial strain E. coli HB101Cre pFIXluc. Bacterial cells were directly added to the PCR mixtures containing primers AR1F and RE2R. (A) A diagram showing the PCR test strategy. (B) An agarose gel electrophoresis indicating absence of amplification of the pGETrec-specific 680 bp DNA segment after pGETrec eviction by the incompatible plasmid pFIXluc. [file 1472-6750-6-17-S3.pdf]

A

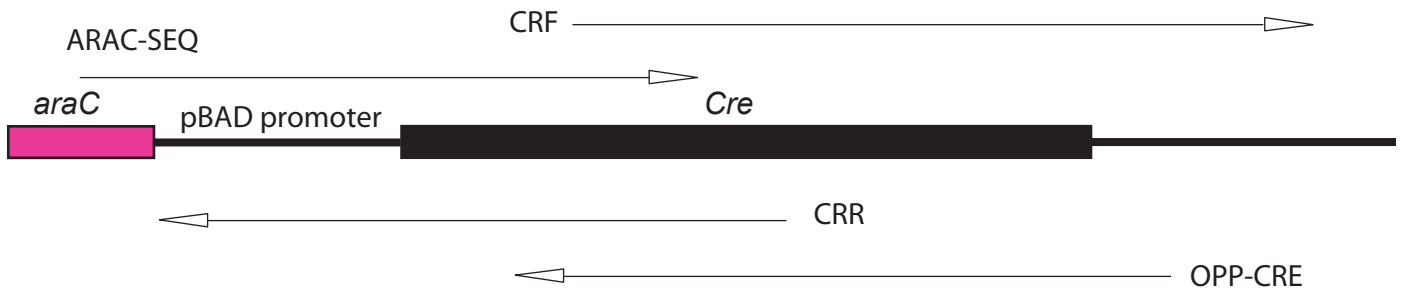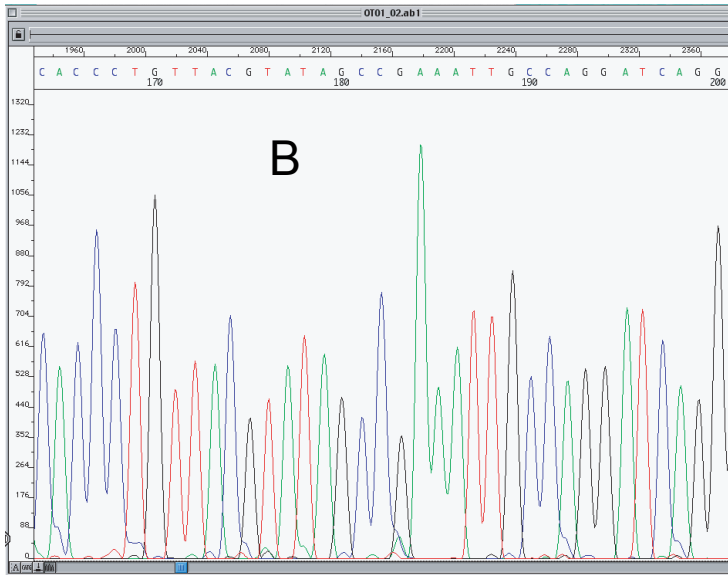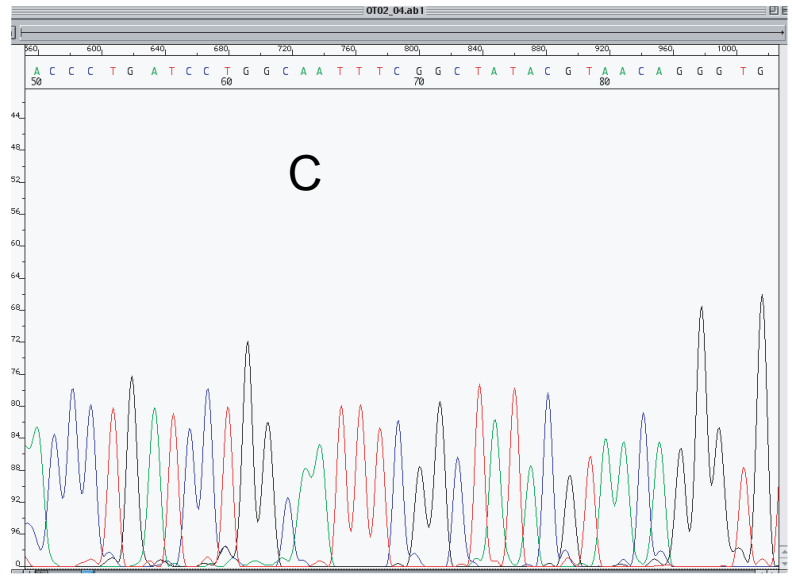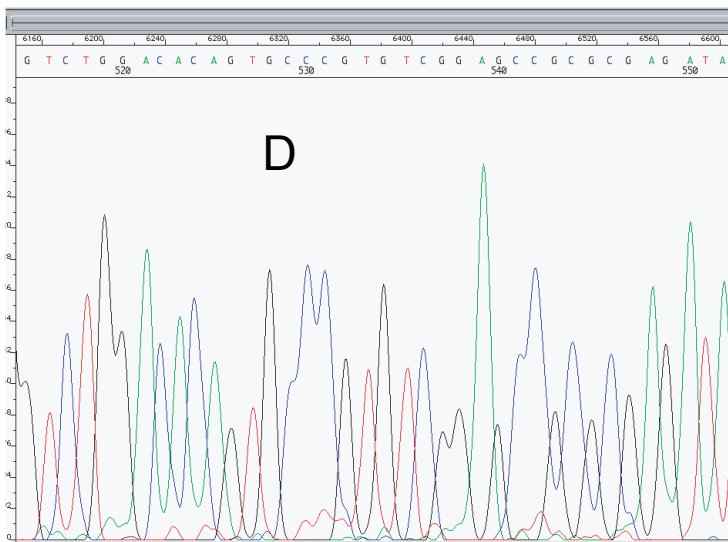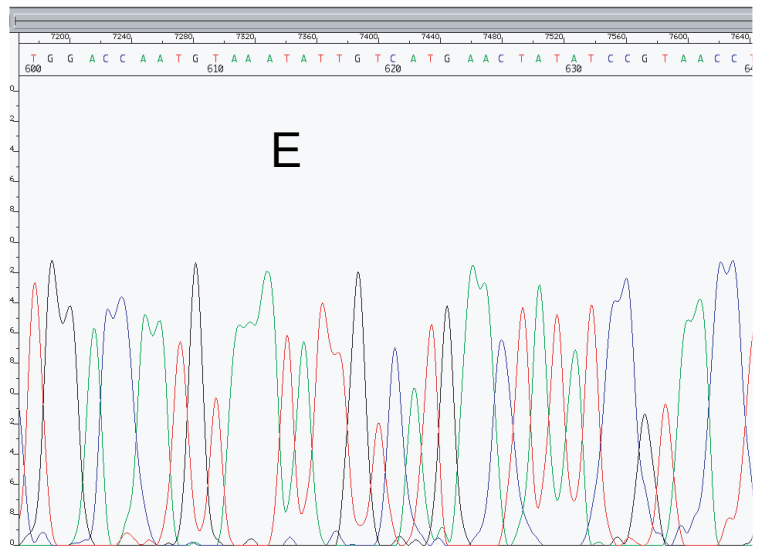

Supplement: Additional File 4 — Sequencing of the Cre gene from the bacterial strain E. coli HB101Cre. Primers LACZ-FA and LACZ2 homologous to the lacZ gene were used to amplify the 3094 bp fragment with the araC-Cre insert in the lacZ gene of the HB101Cre. (A) A diagram showing the strategy to sequence the Cre gene within the amplified 3094 bp DNA fragment. Primers ARAC-SEQ, CRF, CRR and OPP-CRE (5'-GGGCACACAC TACTTGAAGC ACTC-3') were used to sequence the opposite DNA strands. The obtained sequence was shown to be identical to the wild type Cre sequence [GenBank:AF234172]. (B) A part of the electropherogram showing the sequence 5'-ACC CTG TTA CGT ATA GCC GAA ATT GCC AGG ATC AGG-3' of the Cre gene (obtained using primer CRF), which encodes a stretch of amino acids TLLRIAEIARIR including R173 (shown in bold) from the Cre recombinase catalytic site [25]. (C) A part of the electropherogram showing the same sequence as in (B), read from the opposite strand (5'-CCT GAT CCT GGC AAT TTC GGC TAT ACG TAA CAG GGT-3', obtained using primer CRR). (D) A part of the electropherogram showing the sequence 5'-TCT GGA CAC AGT GCC CGT GTC GGA GCC GCG CGA GAT-3' of the Cre gene (obtained using primer CRF), which encodes a stretch of amino acids SGHSARVGAARD including H289 and R292 (shown in bold) from the Cre recombinase catalytic site [25]. (E) A part of the electropherogram showing the sequence 5'-TGG ACC AAT GTA AAT ATT GTC ATG AAC TAT ATC CGT AAC-3' of the Cre gene (obtained using primer CRF), which encodes a stretch of amino acids WTNVNIVMNYIRN including W315 and Y324 (shown in bold) from the Cre recombinase catalytic site [25]. [file 1472-6750-6-17-S4.pdf]

A

luciferase gene

mFIXluc  
3089 bp

From SEQ-CMV  
pCMV loxP66/71

From SEQ-D

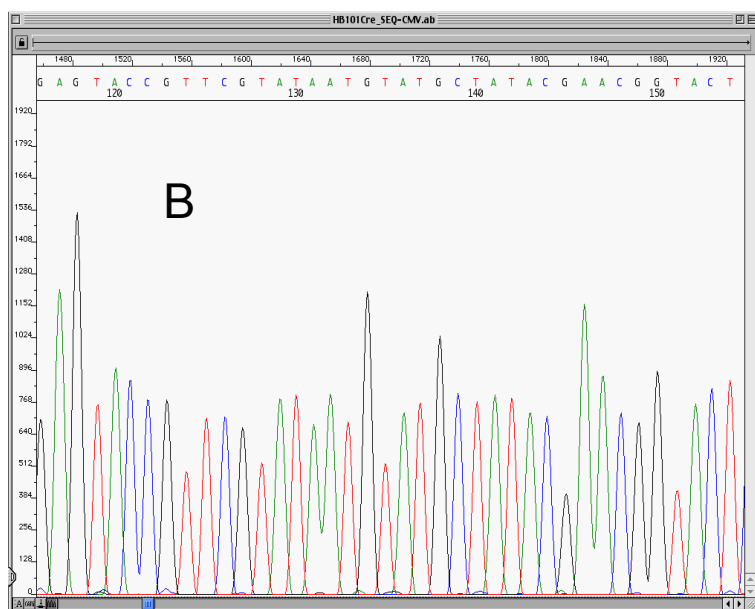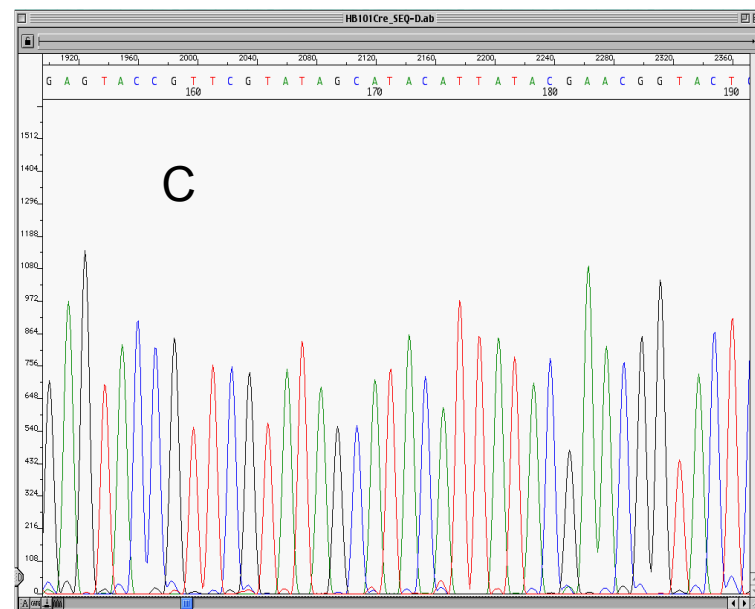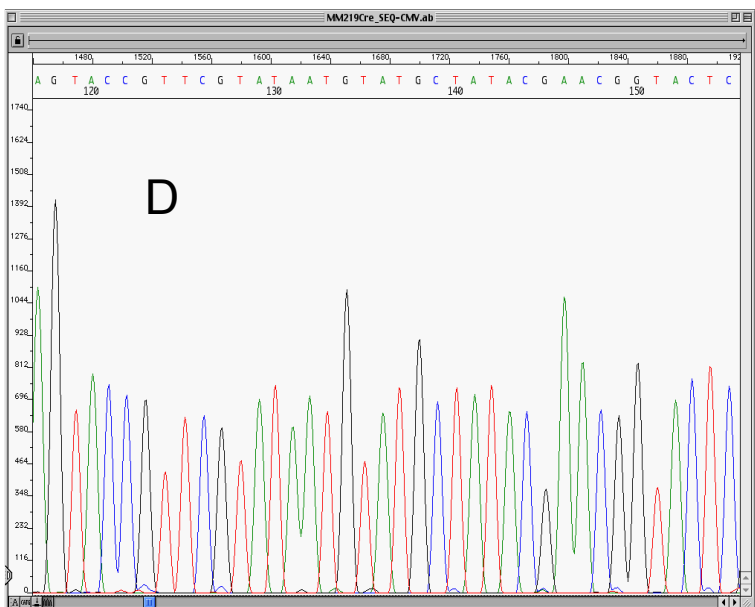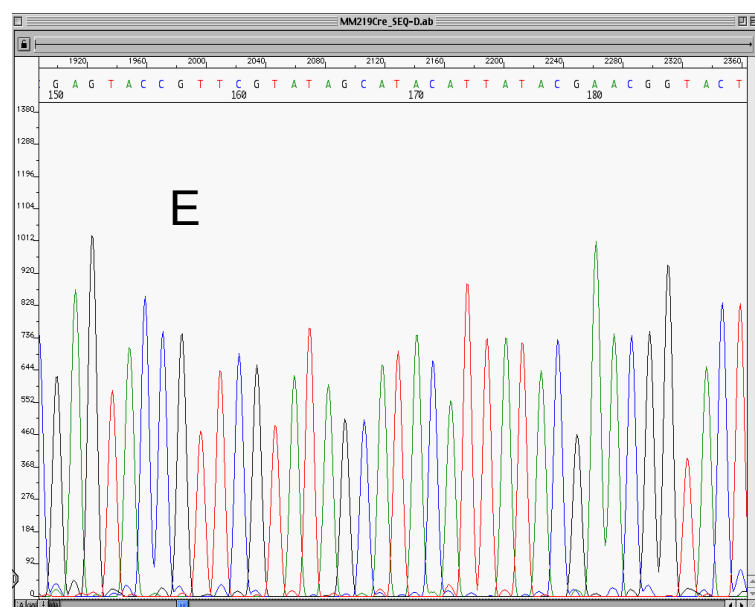

Supplement: Additional File 5 — Sequencing of the minicircle DNA. Minicircle mFIXluc was isolated from the recA- E. coli strain HB101Cre and recA+ E. coli strain MM219Cre, both harbouring the minicircle producer plasmid pFIXluc before L-arabinose induction of Cre recombinase expression. (A) A diagram showing minicircle DNA sequencing strategy. Primers SEQ-CMV and SEQ-D were used to sequence opposite strands of the hybrid loxP66/71 site and the flanking regions. (B) A part of the electropherogram showing the sequence of loxP66/71 (5'-TACCGTTCGT ATAATGTATG CTATACGAAC GGTA-3') of the minicircle mFIXluc extracted from E. coli HB101Cre (SEQ-CMV primed extension). (C) A part of the electropherogram showing the sequence of loxP71/66 (5'-TACC GTTCGTATAG CATACATTAT ACGAACGGTA-3') of the minicircle mFIXluc extracted from E. coli HB101Cre (SEQ-D primed extension). (D) A part of the electropherogram showing the sequence of loxP66/71 site of the minicircle mFIXluc extracted from E. coli MM219Cre (SEQ-CMV primed extension). (E) A part of the electropherogram showing the sequence of loxP71/66 site of the minicircle mFIXluc extracted from E. coli MM219Cre (SEQ-D primed extension). [file 1472-6750-6-17-S5.pdf]
