# Supplementary material for: RecET driven chromosomal gene targeting to generate a RecA deficient Escherichia coli strain for Cre mediated production of minicircle DNA
Source: BMC Biotechnol. 2006 Mar 10;6:17. doi: 10.1186/1472-6750-6-17 (PMC1421399; doi:10.1186/1472-6750-6-17)
Supplement: Additional File 2 — Selection of the bacterial excisants with the araC-Cre insert in the chromosomal lacZΔM15 gene. (A) The passaged bacterial population, which contained the excisants, was plated at 44°C to obtain individual colonies and to encourage the loss of the excised plasmids. Screening of 500 colonies revealed 21 partially Cm-sensitive clones, which were proven to be mixtures of Cm-sensitive (plasmidless) and Cm-resistant (plasmid-bearing) cells. The obtained Cm-sensitive subclones were screened for the presence of the Cre gene by PCR using primers CRF and CRR homologous to the Cre gene sequence and primers LACZ-FA and LACZ2 homologous to the sequences flanking the araC-Cre DNA segment. Bacteria were added directly to the PCR reaction mixtures. (B) Finally, the unwanted plasmid pUC19 was expelled from the Cre-positive clones by the incompatible minicircle producer plasmid pFIXluc maintained by selection for Cm-resistance. [file 1472-6750-6-17-S2.pdf]

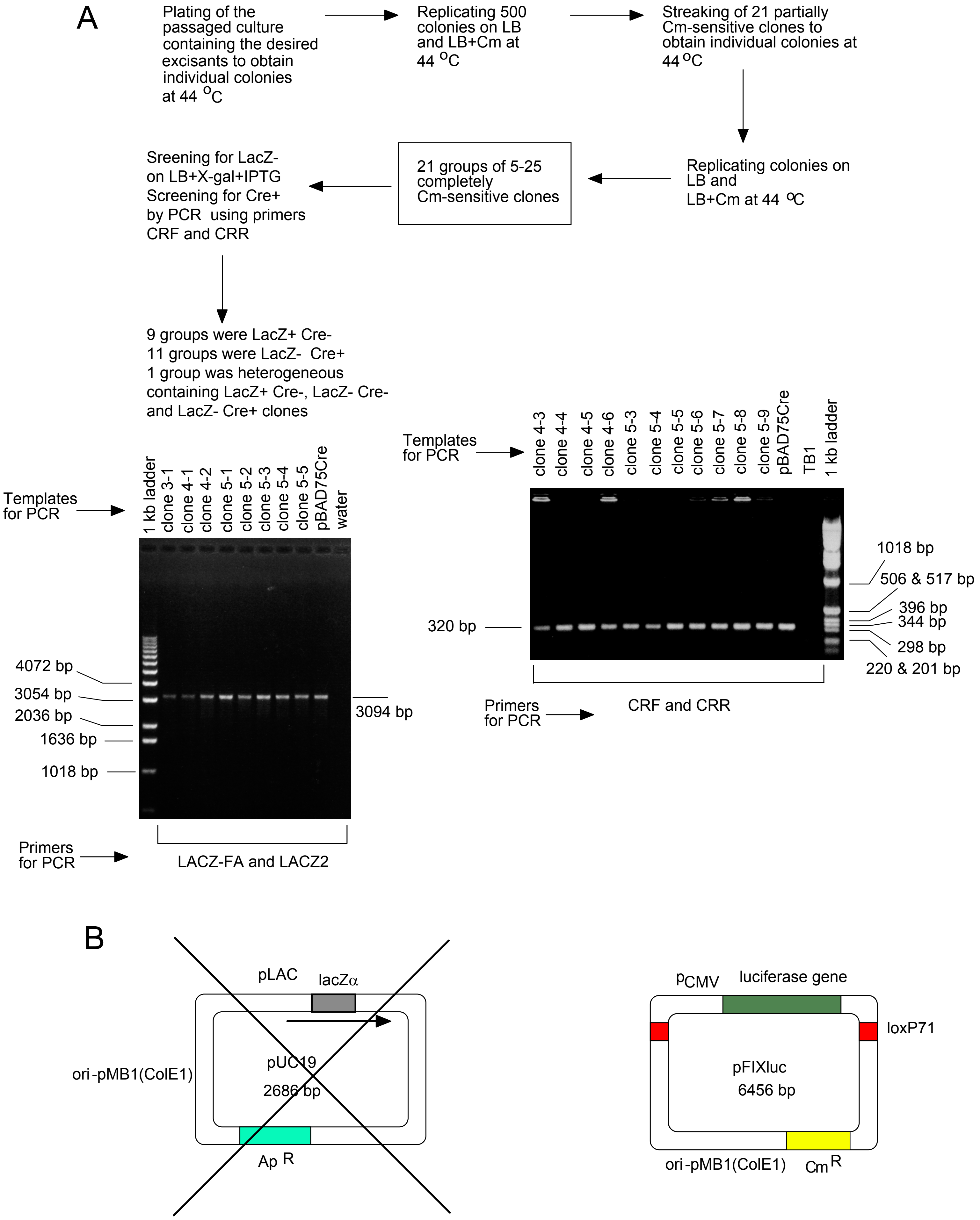

Introduction of minicircle producer plasmid pFIXluc and expulsion of pUC19
